# Rethinking headache in juvenile systemic lupus erythematosus: the need for broader perspectives

**DOI:** 10.1055/s-0046-1817043

**Published:** 2026-03-11

**Authors:** Christian Messina

**Affiliations:** 1 Azienda Sanitaria Provinciale Catania, Catania, Italy.

Dear Editor,

We have read with great interest the study by Cajado et al., 1 who investigated the prevalence, clinical features, and quality-of-life impact of headaches in juvenile systemic lupus erythematosus (jSLE). In this cross-sectional study of 34 patients under 19 years of age, headaches were reported in 50% of the cases, predominantly among female subjects, with a mean age at onset of 11.3 years. 1 Most patients experienced episodic headaches, frequently triggered by stress or physical activity, and almost 90% reported a family history of migraine. 1 Importantly, patients with headaches demonstrated significantly lower quality-of-life scores compared with those without them, underscoring the clinical burden of this symptom in jSLE. 1 However, despite the relevance of this contribution, several limitations and points requiring clarification warrant discussion.

In their study, the authors excluded patients with other chronic diseases, acute infections, or recent pulse therapy with methylprednisolone. However, several additional potential causes of chronic headache should also be considered in the exclusion criteria. For instance, headaches may arise secondary to exogenous substance use, such as excessive caffeine or alcohol consumption, both of which are relevant in adolescent populations. Likewise, lifestyle-related factors—including dehydration and sleep deprivation—are particularly important in this age group, in which irregular routines and insufficient hydration or rest are common and may contribute substantially to headache burden. 2 Furthermore, orgasmic headaches, although less frequently discussed, may occur in adolescents with active sexual lives and should be acknowledged as a potential confounder. 3 Similarly, the initiation of new sports activities during adolescence, especially diving or other activities associated with barometric pressure changes, may also trigger headache episodes. Taken together, these considerations underscore the importance of a more comprehensive exclusion strategy to disentangle primary headache associated with jSLE from other common secondary causes in adolescents.

Another potential confounding factor is bruxism and temporomandibular joint (TMJ) disorders. Bruxism, in particular, is well recognized as a frequent cause of headache and orofacial pain in adolescents, and may occur either as an idiopathic condition or in association with systemic diseases such as SLE, where its prevalence has been reported to reach up to 42.9%. 4 These conditions not only contribute to headache occurrence but also have a significant negative impact on quality of life. 4 Given their relatively high frequency in this age group, and their possible overlap with disease-related manifestations, bruxism and TMJ disorders warrant consideration as potential confounders in the assessment of headache in jSLE patients.

Another limitation of the study is the absence of radiological data, particularly brain magnetic resonance imaging (MRI) correlates. Previous research 5 has shown that a history of severe headache is associated with increased volumes of white-matter hyperintensities, while migraine with aura has been uniquely linked to brain infarcts. This raises the important question of whether the jSLE patients included in the study presented with structural brain lesions, and to what extent these may have influenced both headache expression—especially in migraine—and quality-of-life outcomes. Indeed, a higher burden of white-matter lesions has been consistently associated with reduced cognitive and functional brain performance, suggesting that the integration of neuroimaging data could provide valuable insights into the pathophysiological mechanisms and clinical implications of headache in this population.

In conclusion, while the study by Cajado et al. 1 provides valuable insights into the prevalence and clinical impact of headaches in jSLE, the lack of consideration of common confounders, lifestyle-related triggers, TMJ disorders, and neuroimaging correlates limits the generalizability of the findings. Future studies integrating a broader range of exclusion criteria and radiological assessments may yield a more accurate characterization of headache mechanisms in this vulnerable population. Such efforts are crucial to disentangle disease-specific from secondary causes and to optimize strategies aimed at improving quality of life in adolescents with SLE.
